# Reports of the Committees Appointed by the National Medical Convention of 1846, Which Were Adopted by the Convention of 1847

**Published:** 1847-06

**Authors:** 


### ECLECTIC DEPARTMENT,

### AND SPIRIT OF THE MEDICAL PERIODICAL PRESS.

---

*Reports of the Committees appointed by the National Medical Convention of 1846, which were adopted by the Convention of 1847.*

We shall yield our Eclectic Department entirely to the publication of the reports above referred to, believing that we shall thus meet the wishes of our readers. Although the official proceedings published by the association will contain them, it will probably be some time before that work will be issued, nor will it be likely to reach a large portion of the subscribers to our Journal. The report containing the code of Medical Ethics is necessarily excluded by reason of its length, but it is our present intention to publish this also in some future number. It is quite needless, we feel assured, to commend these reports to the careful perusal of the profession. —Editor Buf. Med. Jour.

*Report on an Uniform and elevated Standard of Requirements for the degree of M. D.*

*Resolved*,—That it is desirable that a uniform and elevated standard of requirements for the degree of M. D. should be adopted by all the Medical Schools in the United States; and that a committee of seven be appointed to report on this subject, at a meeting to be held in Philadelphia on the first Wednesday in May, 1847.

*Committee.*—Drs. Haxwell, Cullen, of Richmond; S. A. Patterson, Manchester, Va.; A. Flint, Buffalo, N. Y.; J. Perkins, Castleton, Vt.; J. A. Wing, Albany, N. Y.; Geo. W. Norris, Philadelphia.

The committee under the fourth resolution of the convention which assembled in New-York in May, 1846, and which has for its object the adoption of a uniform and elevated standard of requirements for the degree of M. D., have had the same under consideration, and beg leave to offer the following Report.

[We omit a portion of this report, not because it is irrelevant, but because it does not bear directly on the resolutions submitted, and it is necessary to economize space.—Ed. Buf. Jour.]

In order to obtain a knowledge of the requirements demanded by the various schools of the country, a letter was addressed to all whose existence was known to your committee, requesting them to furnish the desired information. These consisted of thirty three in number; and answers were received from nineteen, being a fraction more than one-half. In the majority of instances pamphlets were forwarded to the chairman, containing the rules and regulations of the college, together with the branches taught; while from others, letters were received simply stating that two full courses of lectures were demanded, that the candidate must be twenty-one years of age, of good moral character, &c., From this correspondence but little available information was derived. A circular was then dispatched to the different colleges propounding the following qustions, answers to which were respectfully solicited. Nineteen of these circulars were filled up and returned.

State 1*st*. The number of students during the session of 1845–6

2*d*. The number of graduates in 1845–6.

3*d*. The number of charity pupils in 1845–6.

4*th*. The number of professors attached to your shool.

5*th*. The date at which lectures begin and end.

6*th*. The requirements for a degree.

7*th*. Is the inquiry made previous to examination, whether or not these requirements are fulfilled?

8*th*. Is any evidence of having attended a course of clini- instruction necessary to graduation?

8*th*. Is it required of candidates for graduation that they shall have devoted any time to dissection?

The responses to these several questions have been arranged in tabular form, from which the result of the whole correspondence may be ascertained at a glance, and which your committee beg leave to lay before this Convention, as a part of this report. We cannot avoid the expression of our regret, that so many of the colleges have failed to respond to our circular, not, we hope, from a listless indifference to the subject, or a determination not to abide by the recommendations of this body. Information was also requested from the proper authorities in regard to requirements of surgeons in the army and navy of the United States; but no reply has been received from them.*

In summing up the information obtained from the nineteen colleges named in the table, in the order in which the questions were proposed, it will be found that the number of students belonging to these institutions during the session of 1845–6, amounted to 2544, including 31 non-paying pupils; and that the number of graduates was 730:—the number of professors varies from *three* to *eight*. It may be proper to remark that the lowest number here named is attached to the University of Virginia, where the usual branches of medical education are taught, and where the term of study extends throughout the period of *nine* months. If we exclude this institu-

---

* A reply was subsequently received and appended to the report in a note.

tion from our table, the minimum is *five* and the maximum is *eight.* The time employed in lecturing also varies; *thirteen* weeks being the shortest, and *eighteen* the longest period. *Sixteen* weeks, however, are devoted to the lectures, in a large majority of the schools. The requirements for the degree of M. D. appear to be very general;—the candidate must be twenty-one years of age;—his moral character good;—his examination satisfactory;—his thesis passable, and he must have attended two full courses of lectures, to be included within the period of three years' study. In some of the schools a practice of four years, and an attendance on one course of lectures, is sufficient to entitle the individual to an examination. Branches are taught in some institutions which are omitted in others, and the manner in which these are distributed is by no means uniform. It appears without exception that the inquiry is made previous to examination whether or not all the requirements have been fulfilled, and in some cases unquestionable proof of the fact must be adduced. Evidence of having attended a course of clinical instruction is required in *twelve* of the colleges, while in *seven* it is not; and as to dissection, *five* render it obligatory, and the remaining *fourteen* are content to urge its recommendation.

Such, then, is the information which the committee, after diligent inquiry, is enabled to lay before this body. It would have been a matter of some importance to have received answers from all the schools, for in that case an account entirely accurate of the whole number of students in the United States would have been obtained. It will be seen that the nineteen colleges which noticed our circular, report the aggregate number to be 2544; and if it be proper to assign the same ratio to the fourteen which did not, the whole number of students during the session of 1845–6, will bo found to be 4418 and a fraction. We believe this to be a very fair statement, for several statistical tables published from time to time in the journals, have varied but little from this amount; and if we extend the same rule to the classes of graduates, we reach the conclusion that a fraction less than 1300 were added to the already existing number of physicians, in the spring of 1846.

The very large number of physicians in the United States, a number far larger in proportion to its population than in any other country perhaps of which we have a correct knowledge, has frequently been the subject of remark. To relieve the diseases of something more than twenty millions of people, we have an army of Doctors amounting by a recent computation to forty thousand, which allows one to about every five hundred inhabitants. And if we add to the 40,000 the long list of irregular practitioners who swarm like locusts in every part of the country, the proportion of patients will be still further reduced. No wonder, then, that the profession of medicine has measurably ceased to occupy the elevated position which once it did; no wonder that the merest pittancae in the way of remuneration is scantily doled out even to the most industrious in our ranks,—and no wonder that the intention, at one time correct and honest, will occasionally succumb to the cravings of a hard necessity. The evil must be corrected. With a government like ours, to diminish the number of medical schools is not to be expected; and the corrective can alone be found in the adoption of such a standard of requirements, in the general estimate of which the recommendations of this committee will form but an item, as will place the diploma beyond the reach of those who seek to wear its honors without deserving them.

The shortness of the time allotted to the delivery of Lectures, we believe to be an evil of no small magnitude. It is next to an impossibility, that the strongest intellect can receive and well digest some half a dozen discourses or more a day, embracing subjects which have oftentimes little or no immediate connection with each other. The mind becomes wearied with the multiplicity of its occupations, and the thoughts of to-day are forgotten in the constantly recurring and harassing duties of the morrow. A proper allotment of time cannot be given to that deep reflection which the importance of the subject demands, and without which no solid advancement can be made. There are individuals, too, who do not always rest satisfied with professorial prelections, who approve in its fullest extent the adage, "nullius addictus jurare in verba magistri," and who seek in the recorded experience of others, the widest extent of information. Nor can Teachers themselves, with every disposition to impart the fullest instruction, do justice to their several branches within the limited period of four months. For this reason, the examination which may, indeed, test the actual acquirement of the student, can never embrace an extensive field of knowledge. Under the present regulation this cannot be expected; for justice to the pupil must prevent an inquiry into subjects in which he has never been instructed.

In regard to the branches to be taught, it is not improbable that the recommendation of the Committee may not meet the views of some of the members of this Convention. It may be thought that others should have been added, and that consequently the number of Professors should have been considerably augmented. This subject was freely canvassed by the Committee, and the opinion was general that in the commencement of our effort to reform the existing condition of things, too high a standard of requirement ought not to be immediately insisted on. The conclusions to which this Convention may come upon any subject, cannot be forced upon the schools contrary to their wishes. In the advice of this body there exists, however, a moral power which it would be unwise to disregard, a power which, while it seeks to elevate medical requirement, should not impose a single requisition which is not entirely practicable. Besides, it is to be recollected that the decrees of this Convention need not be final and unalterable. The National Medical Association, contemplated under the first two resolutions adopted at the meeting in May last, which it is to be hoped will convene from time to time in different parts of the country, can legitimately exercise the same influence which we now seek to do; and when in after time it may be deemed advisable to add new subjects of instruction, it will not hesitate to express its opinion. Under this view of the matter, your Committee advise that lectures be given on the following branches, in all the Colleges: viz., on the Theory and Practice of Medicine; the Principles and Practice of Surgery; General and Special Anatomy; Physiology and Pathology; Materia Medica; Therapeutics and Pharmacy; Midwifery, and Diseases of Women and Children; Chemistry and Medical Jurisprudence.

These subjects, it is true, are now taught in the majority of our schools. With a lengthened period for teaching, a double advantage will be gained; a wider extent of information may be imparted to the student, while his time will be occupied with fewer lectures during the day. As to the appropriation of the several branches, it is deemed inexpedient to make any suggestion, as the circumstances of each Institution may be such as will necessarily lead to a different arrangement in this particular.—It is believed, however, that a slight augmentation in the number of Professors will become necessary.

In regard to clinical instruction, your Committee would willingly have made it a requisition to the attainment of the diploma; but an insuperable difficulty seemed to exist. It is believed that few of the Colleges have Hospitals attached to them, and in very many instances where such Institutions exist in the cities, the Professors do not receive the appointment of attending Physicians and Surgeons. Consequently, the opportunity is lost to them of imparting such instruction as would be valuable to the student.

We cannot, however, close this portion of our report, without urging the very great importance of clinical instruction. Many a young man has entered upon the duties of his profession, sufficiently instructed, it may be, in its principles, who has most painfully felt the responsibility of his position. In default of a certain degree of practical knowledge, indecision must often fetter his efforts to the detriment of his patient, and that self-confidence, which can alone restore serenity to his mind, must be purchased by a long period of perplexing doubt. During the pupilage of the student, he should lose no opportunity of witnessing cases of disease wherever they are to be found. Instructors themselves, should esteem it their highest duty to introduce their pupils to the bedside of their patients in all cases where propriety will admit of it. The Hospitals, Penitentiaries, and Poor-houses, found in so many of our towns and cities, will furnish from time to time, cases of every description, while the pauper practice of the country will prove no indifferent means of imparting clinical information. Instruction gained from these sources in free and familiar conversation with the Preceptor, will perhaps be of more avail than any other; for the large number of Students who occasionally throng the Wards of the Hospital, render it impossible for each one to obtain a correct knowledge of the cases presented to him. We do not intend by this remark to convey the idea that information given in this way would be valueless. On the contrary, when properly conducted, it may be made productive of the greatest good. Let the Professor daily analyze the symptoms which arise in every case; have them carefully noted down in his ward-book, together with his prescriptions, and at its termination let him review the whole ground, aided by a post-mortem examination if it result unfavorably, and he will impart instruction which is truly valuable.

It will be seen that only five of the colleges insist upon dissection as a requisite to graduation. Your committee accord in the opinion, that all should render it obligatory upon the student to devote some portion of his time to this needful pursuit. To enter into an argument to prove its absolute necessity, not only to the surgeon, but also to the physician, would be a work of supererogation. We therefore offer without further remark, the following resolutions for the adoption of this Convention:

*Resolved*, 1st. That it be recommended to all colleges to extend the period employed in lecturing, from four, to six months.

2d. That no student shall become a candidate for the degree of M. [illegible]., unless he shall have devoted three entire years to the study of medicine, including the time allotted to attendance upon lectures.

3d. That the candidate shall have attended two full courses of lectures, that he shall be twenty-one years of age, and in all cases shall produce the certificate of his preceptor, to prove when he commenced his studies.

4th. That the certificate of no preceptor shall be received who is avowedly and notoriously an irregular practitioner, whether he shall possess the degree of M. D., or not.

5th. That the several branches of medical education already named in the body of this report, be taught in all the colleges; and that the number of Professors be increased to seven.

6th. That it be required of candidates that they shall have *steadily* devoted three months to dissections.

7th. That it is incumbent upon Preceptors to avail themselves of every opportunity to impart clinical instruction to their pupils; and upon Medical Colleges to show that they have attended upon Hospital practice for one session whenever it can be accomplished, for the advancement of the same end.

8th. That it is incumbent upon all schools and colleges granting Diplomas, fully to carry out the above requisitions.

9th. That it be considered the duty of Preceptors, to advise their students to attend only such institutions as shall rigidly adhere to the recommendations herein contained.

All which is most respectfully submitted.

*May* 1847. RO. W. HAXALL, *Chairman.*

***Report of Committee on Preliminary Education of Students of Medicine.***

The duty entrusted to the Committee was "To report on the standard of acquirements which should be exacted of young men, before being received as students of medicine."

Before attempting to perform this duty, the Committee thought it desirable to ascertain, by inquiries addressed to the medical schools and distinguished practititioners of medicine throughout the Union, the sentiments and practice of the profession on this interesting subject. They, accordingly prepared a circular, the object of which was to ascertain the views of the various Medical Schools in regard to the standard of preliminary education which it is proposed to exact of young men about to commence the study of medicine; and also to invite any suggestions they might think proper to make. Copies of this circular were sent to the (then) thirty-six Medical Schools of the United States, and official replies have been received from six of this number; namely, the Medical Department of the University of Pennsylvania, the College of Physicians and Surgeons in the city of New-York, the Medical Institution of Yale College, Ohio Medical College, Albany Medical College, and the Medical Faculty of Dartmouth College. These institutions are desirous of having a standard established, and the Committee do not doubt that they, as well as others, will cheerfully co-operate with the Convention and the profession in this desirable reform.

Another circular was addressed to distinguished medical practitioners residing in all the States and Territories of the Union, soliciting replies to the following questions.

1. Is it the custom of your State or neighborhood to require of young men, who desire to study medicine, any particular amount of preliminary education?

2. If so, what are the details of your standard?

3. By what authority, whether of a State Medical Society, local Association, or common consent, is this standard established?

4. What may be considered the general sentiment of the profession in your vicinity, in regard to the establishment of such a standard as is contemplated in the 5th resolution of the National Medical Convention of May, 1846?

The Committee have been favored with very full and explicit answers to this circular from thirty-nine gentlemen, representing twenty-one States of the Union. The replies which have been received to the first three questions, establish the fact, not only that there is no uniform standard preparatory educution exacted of medical students throughout the United States, but that there is no general rule adopted in any particular state or district, which has been authorized or recommended by Medical Societies or other official bodies, or established by common consent and custom. The whole subject is left to private preceptors, many of whom recommended, and a few exact, an elevated standard, while others leave it to the direction of the students themselves, or their parents. But all the letters of the practitioners indicate, without an exception, the cheering fact, that the profssion is alive to the want of a standard, is desirous that one should be established by the Convention, and is willing to sustain it.

The fourth question was designed to elicit the general sentiment of the profession in regard to the nature and extent of the preliminary education which should be required of medical students. On this subject, the correspondence shows a considerable diversity of opinion; and the standard of different writers varies from a "common school" education up to the highest collegiate attainments. Some advocate a different scale in different sections of the country, while a majority is in favor of having the standard uniform throughout the Union. And, notwithstanding the individual difference of ideas, there is a sufficient general concurrence in the views of the writers to enable the Committee to recommend a scale which is chiefly based upon their united suggestions.

Your Committee are aware of the difficulties the Convention would discover in fixing the standard of preliminary education for medical students as high as would be desirable. Entirely destitute of the means of legal compulsion, and depending for success, as the Convention must, solely upon the force of professional and public opinion, nothing could be hoped from a standard above the circumstances of the country and the times. The existing evil can be reached only by the concurrent action of private medical preceptors, and medical schools of the country. The chief responsibility rests with the preceptors; and to them the Convention should make its strongest appeal; but at the same time they should be encouraged and sustained by the co-operation of the schools, without which, indeed, the efforts of preceptors conld be but partially successful.

The object to which the Committee has directed its labors, it is believed, can be best effected by the Convention in the following way:—

1*st.* By establishing a uniform standard of preliminary education for medical students, which shall be of a moderate character — in the first instance, too low, rather than too high—and yet of such extent as will insure both the knowledge and the mental discipline necessary to those who would enter a profession full of labor and responsibility, without excluding meritorious young men of limited means and opportunities.

2*d.* By earnestly recommending every medical preceptor to exact this standard of every young man, before admitting him into his office; and having exacted it, to grant him a written certificate to that effect, specifying also the period of his admission into the preceptor's office, as a proper warrant and credential for the student, when about entering a medical college.

3*d*. By requesting all the medical colleges of the country to require such a certificate of every student applying for matriculation; and, in publishing their annual lists of graduates, to accompany the name of the graduate with the name and residence of his preceptor, the name of the latter being clearly and distinctly presented as certifying to the qualification of preliminary education.

These ideas the Committee have put into the form of distinct resolutions, which they append to their report, submitting both for the consideration of the Convention, and, if it think proper, its adoption.

1*st*. ***Resolved***, That this Convention earnestly reccommends to members of the medical profession throughout the United States, to satisfy themselves, either by personal inquiry or the written certificate of competent persons, before receiving young men into their offices as Students, that they are of good moral character, and that they have acquired a good English education, a Knowledge of Natural Philosophy and the Elementary Mathematical Sciences, including Geometry and Algebra; and such an acquaintance, at least, with the Latin and Greek languages, as will enable them to appreciate the technical language of medicine, and read and write prescriptions.

2*d*. ***Resolved***, That this Convention also recommends to the members of the medical profession of the United States, when they have satisfied themselves that a young man possesses the qualifications specified in the preceding resolution, to give him a written certificate, stating that fact, and recording also the date of his admission as a medical student, to be carried with him as a warrant for his reception into the medical college in which he may intend to pursue his studies.

3*d*. ***Resolved***, That all the medical colleges in the United States be, and they are hereby recommended and requested to require such a certificate of every student of medicine applying for matriculation; and when publishing their annual lists of graduates, to accompany the name of the graduate with the name and residence of his preceptor, the name of the latter being clearly and distinctly presented as certifying to the qualification of preliminary education.

Signed by

JAMES COUPER,

L. P. BUSH,

JAMES W. THOMPSON,

ALDEN MARCH,

WASHINGTON L. ATLEE,

} Committee.

---

*Report of the Committee on the Organization of the National Medical Association, as ordered by the National Medical Convention held in the city of New-York in the month of May*, 1846.

The Medical Convention held in the city of New-York, in May last, having resolved, "That it is expedient for the Medical Profession of the United States to institute a National Medical Association," and having appointed a committee of seven, consisting of Drs. John Watson, John Stearns, and F. Campbell Stewart, of the city of New-York; A. Stille, of Philadelphia; N. S. Davis, of Binghamton, N. Y.; W. H. Cogswell, of Plainfield, Conn.; and E. D. Fenner, of New-Orleans, "To report a plan of organization for such an association, at a meeting to be held at Philadelphia, on the first Wednesday in May, 1847;" the said committee, after carefully deliberating on the business entrusted to them, beg leave respectfully to Report.

*Plan of Organization for a National Medical Association.*

Whereas, the Medical Convention, held in the city of New-York in May 1846, have declared it expedient "for the Medical Profession of the United States to institute a National Medical Association;" and,

Inasmuch as an institution so conducted as to give frequent, united and emphatic expression to the views and aims of the Medical Profession in this country, must at all times have a beneficial influence, and supply more efficient means than have hitherto been available here, for cultivating and advancing medical knowledge, for elevating the standard of medical education, for promoting the usefulness, honor, and interests of the Medical Profession; for enlightening and directing public opinion in regard to the duties, responsibilities and requirements of medical men. for exciting and encouraging emulation and concert of action in the profession, and for facilitating and fostering friendly intercourse between those who are engaged in it;—therefore

Be it resolved, in behalf of the Medical Profession of the United States,—that the members of the Medical Convention held in Philadelphia, in May, 1847, and all others who in pursuit of the objects above-mentioned, are to unite with, or succeed them, constitute a National Medical Association;—and that, for the organization and management of the same, they adopt the following Regulations.

*I. Title of the Association.*—This institution shall be known and distinguished by the name and title of "The American Medical Association."

*II. Members.*—The members of this institution shall collectively represent and have cognizance of the common interests of the medical profession in every part of the United States; and shall hold their appointment to membership either as delegates from local institution, as members by invitation, or as permanent members.

*The delegates* shall receive the appointment from permanently organized medical societies, medical colleges, hospitals, lunatic asylums, and other permanently organized medical institutions of good standing, in the United States. Each delegate shall hold his appointment for one year, and until another is appointed to succeed him, and shall participate in all the business and affairs of the association.

Each local society shall have the privilege of sending to the association one delegate for every ten of its regular resident members, and one for every additional fraction of more than half of this number. The faculty of every regularly constituted medical college or chartered school of medicine, shall have the privilege of sending two delegates. The professional staff of every chartered or municipal hospital containing a hundred inmates or more, shall have the privilege of sending two delegates; and every other permanently organized medical institution of good standing shall have the privilege of sending one delegate.

*The Members by Invitation* shall consist of practitioners of reputable standing, from sections of the United States not otherwise represented at the meeting. They shall receive their appointment by invitation of the meeting after an introduction from any of the members present, or from any of the absent permanent members. They shall hold their connection with the association until the close of the annual session at which they are received; and shall be entitled to participate in all its affairs, as in the case of delegates.

*The Permanent Members* shall consist of all those who have served in the capacity of delegates, and of such other members as may receive the appointment by unanimous vote.

Permanent members shall at all times be entitled to attend the meetings, and participate in the affairs of the association, so long as they shall continue to conform to its regulations; and when not in attendance, they shall be authorized to grant letters of introduction to reputable practitioners of medicine residing in their vicinity, who may wish to participate in the business of the meetings, as provided for members by invitation.*

Every member elect, prior to the permanent organization of the annual meeting, or before voting on any question after the meeting has been organized, must sign these regulations, inscribing his name and address in full, specifying in what capacity he attends, and, if a delegate, the title of the institution from which he has received his appointment.

*III. Meetings.*—The regular meetings of the Association shall be held annually, and commence on the first Tuesday of May. The place of meeting shall never be the same for any two years in succession. and shall be determined for each next succeeding year by vote of the Association.

*IV. Officers.*—The officers of the Association shall be a President, four Vice Presidents, two Secretaries, and a Treasurer. They shall be nominated by a special committee of one member from each state represented at the meeting, and shall be elected by vote on a general ticket. Each officer shall hold his appointment for one year, and until another is elected to succeed him.

*The President* shall preside at the meetings, preserve order and decorum in debate, give a casting vote when necessary, and per-

*By an amendment adopted, permanent members are not permitted to vote.

form all the other duties that custom and parliamentary usage may require.

*The Vice Presidents*, when called upon, shall assist the President in the performance of his duties, and, during the absence, or at the request of the president, one of them shall officiate in his place.

*The Secretaries* shall record the minutes, and authenticate the proceedings, give due notice of the time and place of each next ensuing annual meeting, and serve as members of the Committee on Publication. The Secretary first in nomination shall also preserve the archives and unpublished transactions of the Association.

*The Treasurer* shall have the immediate charge and management of the funds and property of the Association. He shall be a member of the Committee on Publication, to which committee he shall give bonds for the safe keeping, and proper use, and disposal of his trust. And through the same committee he shall present his accounts, duly authenticated, at every regular meeting.

*V. Standing Committees*.—The following Standing Committees, each composed of seven members, shall be organized at every annual meeting, for preparing, arranging, and expediting business for each next ensuing year, and for carrying into effect the orders of the Association not otherwise assigned—namely, a Committee on Arrangements, a Committee on Medical Sciences, a Committee on Practical Medicine, a Committee on Surgery, a Committee on Obstetrics a Committee on Medical Education, a Committee on Medical Literature, and a Committee on Publication.

*The Committee on Arrangements* shall, if no sufficient reasons prevent, be mainly composed of members residing in the place at which the Association is to hold its next annual meeting; and shall be required to provide suitable accommodations for the meeting, to verify and report upon the credentials of membership, to receive and announce all essays and memoirs voluntarily communicated, either by members of the Association, or by others through them, and to determine the order in which such papers are to be read and considered.

*The Committee of Medical Sciences* shall prepare an annual report on the progress of Medical Sciences in America, noticing, as occasion may require, the more important improvements and discoveries in Anatomy, Physiology, Hygiene, General Pathology and Therapeutics, Medical Jurisprudence, Materia Medica, and other branches of natural science, bearing directly on the condition and progress of medical knowledge in America, during the year of their service.

*The Committee on Practical Medicine* shall prepare an annual report on the more importont improvements effected in this country in the management of individual diseases; and on the progress of epidemics: referring, as occasion requires, to medical topography, and to the character of prevailing diseases in special localities, or in the United States generally, during the term of their service.

*The Committee on Surgery* shall prepare an annual report on all the important improvements in the management of surgical diseases effected in America during the year.

*The Committee on Obstetrics* shall prepare an annual report on all the important improvements in the Obstetric Art, and in the management of diseases peculiar to women and children, effected in America during the year.

*The Committee on Medical Education* shall prepare an annual report on the general condition of medical education in the United States, in comparison with the state of medical education in other enlightened nations; noticing, as occasion may call for, the courses of instruction, the practical requirements for graduation, the modes of examination for conferring degrees, and the reputed number of pupils and of graduates at the several medical institutions in the United States, during the year:—noticing also the requirements of the United States Army and Navy Boards of Medical Examiners, the legal requirements exacted of medical practitioners in our several states, and all such measures, prospective or established, in reference to medical education and the reputable standing of the profession, as may be deemed worthy of special consideration.

*The Committee of Medical Literature* shall prepare an annual report on the general character of the periodical medical publications of the United States, in reference to the more important articles therein presented to the Profession, on original American medical publications, on medical compilations and compends by American writers, on American reprints of foreign medical works and on all such measures as may be deemed advisable for encouraging and maintaining a national literature of our own.

*The Committee on Publication*, of which the Secretaries and Treasurer must constitute a part, shall have charge of preparing for the press, and of publishing and distributing such of the proceedings, transactions and memoirs of the Association, as may be ordered to be published. The six members of this Committee, who have not the immediate management of the funds, shall also in their own names as agents for the Association, hold the bond of the Treasurer for the faithful execution of his office, and shall annually audit and authenticate his accounts, and present a statement of the same in the annual report of the Committee, which report shall also specify the character and cost of the publications of the Association during the year, the number of copies still at the disposal of the meeting, the funds on hand for further operations, and the probable amount of the Assessment to be laid on each member of the Association for covering its annual expenditures.

*VI. Funds and Appropriations.*—Funds shall be raised by the Association for meeting its current expenses and awards from year to year; but never with the view of creating a permanent income from investmonts. Funds may be obtained by an equal assessment of not more than three dollars annually on each of the members; by individual voluntary contributions for specific objects; and by the sale and disposal of publications, or of works prepared for publication.

The funds may be appropriated for defraying the expenses of the annual meetings; for publishing the proceedings, memoirs, and transtions of the association; for enabling the standing committees to fulfil their respective duties, conduct their correspondence, and procure the materials necessary for the completion of their stated annual reports; for the encouragement of scientific investigations, by prizes and awards of merit; and for defraying the expenses incidental to specific investigations under the instruction of the association, where such investigations have been accompanied with an order on the treasurer to supply the funds necessary for carrying them into effect.

*VII. Provision for Amendments.*—No amendment or alteration shall be made in any of these articles, except at the annual meeting next subsequent to that at which such amendment or alteration may have been proposed; and then only by the voice of three-fourths of all the members in attendance.

And, in acknowledgment of having adopted the foregoing propositions, and of our willingness to abide by them, and use our endeavors to carry into effect the objects of the association, as above set forth,—we have hereunto affixed our names.

| NAMES OF MEMBERS. | RESIDENCE. | INSTITUTIONS REPRESENTED. |
| --- | --- | --- |

In connection with the foregoing "Plan of Organization," the committee beg leave further to report the following as one of the ordinances, or by-laws of the proposed association, viz:

*The Order of Business.*—The order of business at the annual meetings of the American Medical Association shall at all times be subject to the vote of three-fourths of all the members in attendance; and until permanently altered, except when for a time suspended, it shall be as follows, viz:

1. The temporay organization of the meeting preparatory to the election of officers.

2. The report of the committee of Arrangements on the credentials of members; after the latter have registered their names and addresses, and the titles of the institutions which they represent.

3. The calling of the roll.

4. The election of officers.

5. The reading of minutes.

6. The reception of members not present at the opening of the meeting, and the reading of notes from absentees.

7. The reception of members by invitation.

8. The reading and consideration of the stated annual reports from the standing committees.

9. The selection of the next place of annual meeting.
10. The dew appointments to fill the standing committees.
11. The choice of permanent members by vote.
12. Resolutions introducing new business, and instructions to the permanent committees.
13. The reading and discussion of voluntary communications introduced through the committee on arrangements.
14. Unfinished and miscellaneous business.
15. Adjournment.

Before bringing this report to a close, the committee beg leave to remark, that in preparing the foregoing "plan of organization," and "ordinance" accompanying it, they have constantly had in view, as worthy of imitation, the plan of organization and order of business adopted without any previous concert of action, by the Medical Convention of 1846.

The plan here presented, differs from the mode of organization adopted by the late Convention, principally in such points as are necessary to give permanency and influence to the proposed association. It is believed to be sufficiently simple, and at the same time sufficiently comprehensive and practicable, for organizing the whole of the Medical Profession of the United States into a permanent body; and for carrying into effect all the objects contemplated in the formation of a National Medical Association.

All of which is respectfully submitted.

JOHN WATSON.

JOHN STEARNS.

F. CAMPBELL STEWART.

ALFRED STILLE.

N. S. DAVIS.

* E. D. FENNER, (by the Chairman.)

* The remaining reports of committees are reserved for the July No.

